# Influence of Compounding Parameters on Color Space and Properties of Thermoplastics with Ultramarine Blue Pigment

**DOI:** 10.3390/polym15244718

**Published:** 2023-12-15

**Authors:** Puay Keong Neo, Yuki Kitada, Jakawat Deeying, Supaphorn Thumsorn, Moi Fuai Soon, Qing Sheng Goh, Yew Wei Leong, Hiroshi Ito

**Affiliations:** 1 Graduate School of Organic Materials Science, Yamagata University, 4-3-16 Jonan, Yonezawa 992-8510, Yamagata, Japan; pkneo@ops-sys.com; 2 Omni-Plus System Limited, 994 Bendemeer Road, 01-03 B-Central, Singapore 339943, Singapore; mfsoon.sg@ops-sys.com (M.F.S.); qsgoh.sg@ops-sys.com (Q.S.G.); 3 Department of Systems Innovation, Faculty of Engineering, Yamagata University, 4-3-16 Jonan, Yonezawa 992-8510, Yamagata, Japan; 4 Logistics and Supply Chain Management Research Center, Science and Technology Research Institute, King Mongkut’s University of Technology North Bangkok, 1518 Pracharat 1 Road, Wongswang, Bangsue, Bangkok 10800, Thailand; jakawat.deeying@gmail.com; 5 Research Center for GREEN Materials and Advanced Processing, Yamagata University, 4-3-16 Jonan, Yonezawa 992-8510, Yamagata, Japan; 6 Matwerkz Technologies Pte Ltd., 994 Bendemeer Road, 01-03 B-Central, Singapore 339943, Singapore; enquiry@matwerkz.com

**Keywords:** color space, compound, dispersion, masterbatch, thermoplastic, ultramarine blue pigment, ANOVA

## Abstract

The incorporation of thermoplastics with pigments imparts diverse aesthetic qualities and properties to colored thermoplastic products. The selection of pigment type and content, along with specific processing conditions, plays a pivotal role in influencing color properties and overall product performance. This study focuses on optimizing these parameters to ensure the desired color quality and product functionality. Two types of polypropylene copolymer (PPCP) with different melt flow rates (MFRs) and acrylonitrile butadiene styrene (ABS) were compounded with ultramarine blue pigment masterbatch (MB) in concentrations ranging from 1 to 5 wt.% using a twin-screw extruder. The compounding process was conducted at a constant screw speed of 200 rpm and a die temperature of 210 °C. The effects of screw speed and die temperature were investigated at a constant MB of 3 wt.%. Colored samples were fabricated by injection molding. Microscopic analysis revealed a well-dispersed pigment within the PPCP matrix when using the MB. Rheological properties, assessed through the power law index, confirmed effective pigment dispersion, facilitated by shear thinning behavior and controlled shear rate via the manipulation of screw speed and die temperature. The effects of masterbatch contents and processing conditions on color spaces were evaluated using CIELAB and CIELCH, with one-way ANOVA employed to identify statistical significance. Higher opacity in high-MFR PPCP and ABS resulted in increased lightness and color strength, surpassing low-MFR PPCP by 15–40% at equivalent MB contents. Masterbatch content emerged as a significant factor influencing the color spaces of all colored thermoplastics. Further analysis, including Fisher pairwise comparisons of one-way ANOVA, revealed that screw speed influenced the redness and hue of low-MFR PPCP, whereas die temperature affected the lightness and hue of high-MFR PPCP and ABS. Interestingly, the blueness and chroma of colored thermoplastics were minimally affected by both screw speed and die temperature. Notably, regardless of processing conditions, the flexural properties of colored thermoplastics remained comparable to the neat polymer when incorporated with ultramarine blue pigment masterbatch.

## 1. Introduction

Polymer coloring entails the synergistic incorporation of colorants and polymers to enhance aesthetics and value-added across diverse applications. A pigment is one of the colorants that is incorporated with thermoplastics in the polymer coloring [1,2,3,4,5,6,7,8,9]. Characterized by their insolubility and solid composition, pigment particles within the polymer matrix exhibit aggregate formations with irregular shapes. This leads to the formation of agglomerates during compounding, impacting the subsequent color appearance and performance of thermoplastic coloration [1]. Hence, a dispersion process is imperative to break down these agglomerates and enhance the wetting of pigments within the polymer matrix [1,2,10,11].

Masterbatch technology is an alternative way to overcome dispersion in polymer coloring [2,12]. Masterbatch serves as a practical formulation, combining high concentrations of pigments with a carrier and dispersing agent. This hybridization offers a convenient form of colorant application in thermoplastic coloring [2,10,11,12,13,14,15]. The carrier and dispersing agent must exhibit compatibility with the matrix resin while concurrently facilitating the breakdown of agglomerates and preventing the re-agglomeration of pigment particles [10,13,16]. Failure to achieve adequate pigment dispersion can manifest as fluctuations in color intensity, deviations in hue, surface inhomogeneity, and a decline in mechanical performance. The dispersion process is important in ensuring the uniformity and optimal performance of thermoplastic coloration, as highlighted in [1,2,10,11,12,13].

The extrusion process serves as a primary method for polymer coloration. Shear force, viscosity, and residence time are considered to ensure effective material interaction and compatibility, ultimately leading to the achievement of pigment dispersion during compounding within the extruder [8,16]. Key operational variables include screw speed, processing temperature, throughput impact polymer viscosity, the wetting process, and the breakdown of agglomerates, all crucial aspects for optimal pigment dispersion [16]. Therefore, by optimizing extrusion conditions, it is possible to effectively manage pigment dispersion to control color properties and overall product performance.

Polypropylene copolymer (PPCP) and acrylonitrile butadiene styrene (ABS) stand out as robust thermoplastics extensively employed in diverse applications, including automotive components, electrical appliances, home furniture, insulators, pipes, transport containers, and indoor decoration [5,9,17,18,19]. Both organic and inorganic pigments serve as essential colorants for PPCP and ABS [1]. Notably, organic pigments, such as those discussed in the literature [3,6,7], are recognized as nucleating agents for polypropylene, exerting a profound influence on polymer crystallization. This impact is manifested in shrinkage during injection molding [3] and variations in coloring efficiency [7]. Specific organic pigments, like quinacridone red and phthalocyanine blue, have been observed to promote polypropylene crystallization, thereby enhancing impact resistance and tensile properties [6,7], underscoring the role of pigments in the material properties of PPCP. Conversely, inorganic pigments exhibit a contrasting behavior, demonstrating no significant effect on injection mold shrinkage and maintaining stability throughout the compounding and usage phases [1,3]. Ultramarine blue, an inorganic pigment, distinguishes itself as a secure, non-hazardous, and environmentally benign coloring agent, finding widespread application in injection molding processes [20]. In contrast, the use of phthalocyanine blue, an organic pigment, is deemed unsuitable due to its adverse impact on product shrinkage [1]. Ultramarine blue exhibits excellent light fastness and withstands temperatures within the range of 300 to 400 °C, making it a versatile choice in thermoplastics coloration [1].

Consequently, this study focuses on the development of a masterbatch incorporating ultramarine blue pigments for effective dispersion in PPCP, ABS, and other thermoplastics for coloring applications. The formulation of the ultramarine blue pigment masterbatch holds significant promise in achieving optimal pigment dispersion, ensuring a streamlined color-matching process, reducing waste and cleaning costs, and facilitating automated material metering [1,11,12]. Furthermore, the establishment of standards in polymer composition, colorants, and molding conditions for polymer coloration enables predictive insights into polymer morphology, color properties, and overall product performance [1]. It is imperative to recognize that color, being a subjective human perception, can be quantified using instruments such as a spectrophotometer or colorimeter, which facilitates effective communication and quality control in colored product manufacturing [14,21,22,23].

This study aims to elucidate the impact of the ultramarine blue pigment masterbatch and compounding conditions on the color performance of thermoplastics, specifically polypropylene copolymer and acrylonitrile butadiene styrene. Morphology and rheological properties were investigated in the stage of pigment dispersion and microstructure at various compounding conditions. The research systematically explores the influence of masterbatch contents and compounding conditions on color spaces by a spectrophotometer and supports the findings through statistical analysis via analysis of variance (ANOVA). The crystallinity and opacity of the thermoplastics on color properties were also focused. Flexural properties are examined to confirm the retention of mechanical performance in thermoplastics compounded with the ultramarine blue pigment masterbatch.

## 2. Materials and Methods

### 2.1. Materials

Two types of high-impact polypropylene copolymer (PPCP) including Cosmoplene^®^ AW564 (The Polyolefin Company (Singapore) Pte. Ltd., Singapore) and Achieve^TM^ Advanced PP8285E1 (ExxonMobil Asia Pacific Pte. Ltd., Singapore). Acrylonitrile butadiene styrene (ABS, Penang, Malaysia), Toyolac^TM^ 100-322, was supplied by Toray Plastics (Penang, Malaysia) Sdn. Berhad, Penang, Malaysia. The masterbatch (MB) utilized in this study, derived from ultramarine blue pigment compounded with each polymer as a carrier, was provided by Omni-Plus System Limited, Singapore, and Nihon Pigment Sdn. Bhd. (Selangor, Johor, Malaysia). These masterbatches underwent thorough development and modification by the manufacturers to ensure optimal distribution and dispersion of the pigment and compatibility within each polymer matrix. The polymers and masterbatch resins were used as received. Table 1 tabulated melt flow rate (MFR), glass transition temperature (T_g_), melting temperature (T_m_), degradation temperature (T_d_), and residual of solid contents of materials used in this research.

### 2.2. Compounding and Fabrication

PPCP, ABS, and MB were compounded in a twin-screw extruder (KZW5TW-30MG-NH, Technovel Co., Ltd., Osaka, Japan, L/D of screw = 45). The masterbatch contents were varied at 0–5 wt.%. The barrel temperatures were set at 180–230 °C with a screw speed of 200 rpm and a constant throughput of 0.8 kg/h. The effects of die temperature and screw speed on properties were studied at the MB content of 3 wt.%. The screw speeds were varied at 100, 200, and 300 rpm with a die temperature of 210 °C. The barrel temperatures were set at 180–230 °C depending on the die temperatures, which were varied at 190, 210, and 230 °C with the screw speed of 200 rpm. Formulations and conditions are tabulated in Table 2. The MB content of 2 wt.% (MB2) was compounded for comparison study only in ABS.

**Figure 1:**
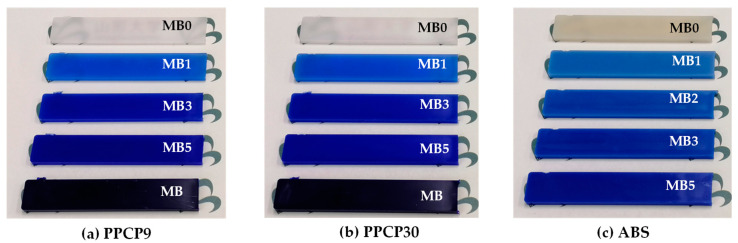
Photographs of injection molded thermoplastics with ultramarine blue masterbatch at 0 to 5 wt.% (MB0 to MB5) and MB (PPCP9) and MB (PPCP30): (**a**) PPCP9; (**b**) PPCP30; (**c**) ABS.

After pelletized, the compounds were injection molded to a bar sample of 2 mm thick, 10 mm wide, and 55 mm long by an 18-ton injection molding machine (iM-18, Sumitomo Heavy Industries, Ltd., Tokyo, Japan). The temperatures were set at 170–220 °C with the first and the holding pressure of 27.8 MPa, and the total injection pressure of 145–185 MPa. The injection speed was set at 30 mm/s. The molding temperature was 60 °C. The injection time and the cooling time were 15 s. Figure 1 shows photographs of injection molded colored thermoplastics with various contents of ultramarine blue masterbatch and the MB of PPCP9 and PPCP30.

In addition, the compounds were hot pressed to 2 mm thick sheets for rheology testing by a compression molding machine (Mini Test Press MP-WC, Toyo Seiki Seisaku-sho, Ltd., Tokyo, Japan) at a temperature of 200 °C with a pressure of 5 MPa for 2 min and cooling at a temperature of 60 °C for 5 min.

### 2.3. Characterization

#### 2.3.1. Morphology

Scanning electron microscope (SEM, TM3030Plus, Hitachi High-Technologies Corporation, Tokyo, Japan) and energy dispersive X-ray spectrometer (EDS, QUANTAX 70, Bruker Japan K.K., Tokyo, Japan) were used to observe the dispersion of ultramarine blue pigment on the surface of injection molded colored thermoplastics. The secondary electron images and EDS element mapping were analyzed.

#### 2.3.2. Rheological Properties

The rheological properties (small-amplitude oscillatory shear, SAOS) of the compression molded samples were analyzed by a rotary rheometer (Modular Compact Rheometer, MCR 302, Anton Paar GmbH, Graz, Austria) by using a 25 mm parallel plate. The temperature was carried out at 190, 210, and 230 °C with an oscillation frequency range of 0.01 to 100 rad/s and a strain rate of 1.0–4.0% for the linear viscoelastic of each material.

#### 2.3.3. Thermal Properties

Thermal decomposition and residuals of polymers, masterbatch, and the injection molded samples were characterized by a thermogravimetric analyzer (TGA, Q50, TA Instruments, New Castle, DE, USA). The sample weight was about 10 mg. The sample was heated from room temperature to 700 °C at a heating rate of 10 °C/min under a nitrogen atmosphere.

Thermal properties and crystallization behavior were investigated by differential scanning calorimeter (DSC, Q200, TA Instruments, New Castle, DE, USA). The sample of about 3–5 mg was sealed in an aluminum pan by using DSC modulation (MDSC) mode at ±1 °C every 60 s. The temperature range was set at 25–200 °C. The heating and cooling rates were 3 and 5 °C/min, respectively [24,25,26], under a nitrogen atmosphere.

#### 2.3.4. Color Measurement

The color space of the bar samples was measured using D65 illuminant at 10° observations (D65/10°) by a benchtop spectrophotometer (Ci7800, X-Rite Incorporated, Michigan, MI, USA). Color indices of CIELAB and CIELCH including lightness ($L^{*}$) index, red/green ($a^{*}$) index, yellow/blue ($b^{*}$) index, chroma ($C^{*}$) index, hue ($h^{{}^{\circ}}$), and reflectance spectrum were recorded using Color iMatch software version 10.7.2. The color difference ${(∆E}_{ab}^{*})$ was calculated from the following equation [7,12,22].

(1) $${∆E}_{ab}^{*}=\sqrt{{\left({\Delta L}^{*}\right)}^{2}{+{\left({\Delta a}^{*}\right)}^{2}+(\Delta b^{*})}^{2}}$$

where $∆L^{*}=L_{2}^{*}-L_{1}^{*}$ is the difference in lightness between the sample $\left(L_{2}^{*}\right)$ and the reference or the standard $\left(L_{1}^{*}\right)$.

$∆a^{*}=a_{2}^{*}-a_{1}^{*}$ is the difference in red or green between the sample $\left(a_{2}^{*}\right)$ and the reference or the standard $\left(a_{1}^{*}\right)$.

$∆b^{*}=b_{2}^{*}-b_{1}^{*}$ is the difference in yellow or blue between the sample $\left(b_{2}^{*}\right)$ and the reference or the standard $\left(b_{1}^{*}\right)$.

#### 2.3.5. Flexural Properties

Flexural properties of injection molded colored thermoplastics were carried out by a universal testing machine (Strograph VGS1-E, Toyo Seiki Seisaku-sho, Ltd., Tokyo, Japan). The span length was 32 mm. The testing speed was 2 mm/min.

## 3. Results and Discussion

### 3.1. Observation of Ultramarine Blue Pigment

**Figure 2:**
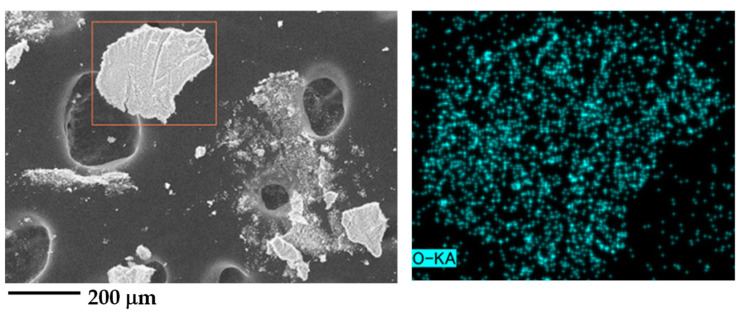
SEM images (**left**) and EDS mapping oxygen element (**right**) at observation area (orange square in SEM image) of ultramarine blue pigment.

The ultramarine blue masterbatch was compounded with PPCP and ABS at various concentrations and under different compounding conditions. The distribution and dispersion of the ultramarine blue pigment were analyzed using scanning electron microscopy (SEM) and energy dispersive spectrometry (EDS). Given that PPCP and ABS thermoplastics exhibit a complex copolymer phase structure, observing the pigment within the polymer matrix may be constrained. In contrast, the chemical composition of the ultramarine blue pigment is represented as Na_8-10_Al_6_Si_6_O_24_S_2-4_, which can be investigated and identified by using EDS [27,28,29]. The EDS mapping of the pigment and PPCP9-colored samples revealed the presence of Na, Al, Si, O, S, and C, enabling the identification of ultramarine blue in the samples [15,28]. The selected elements were prominently observed in the mapping in the pigment, neat PPCP9MB0, the PPCP9MB3, and the MB as depicted in Supplementary Figures S1–S4. Consequently, the EDS mapping primarily focused on the oxygen element to elucidate the relationship between the ultramarine blue pigment and the thermoplastic matrix, confirming the distribution and dispersion of the pigment in the polymer matrix [28]. Figure 2 shows the SEM image (left) and EDS mapping of the oxygen element (right) of the ultramarine blue pigment post-polymer combustion from TGA. The identification of the pigment particles is facilitated through oxygen mapping.

**Figure 3:**
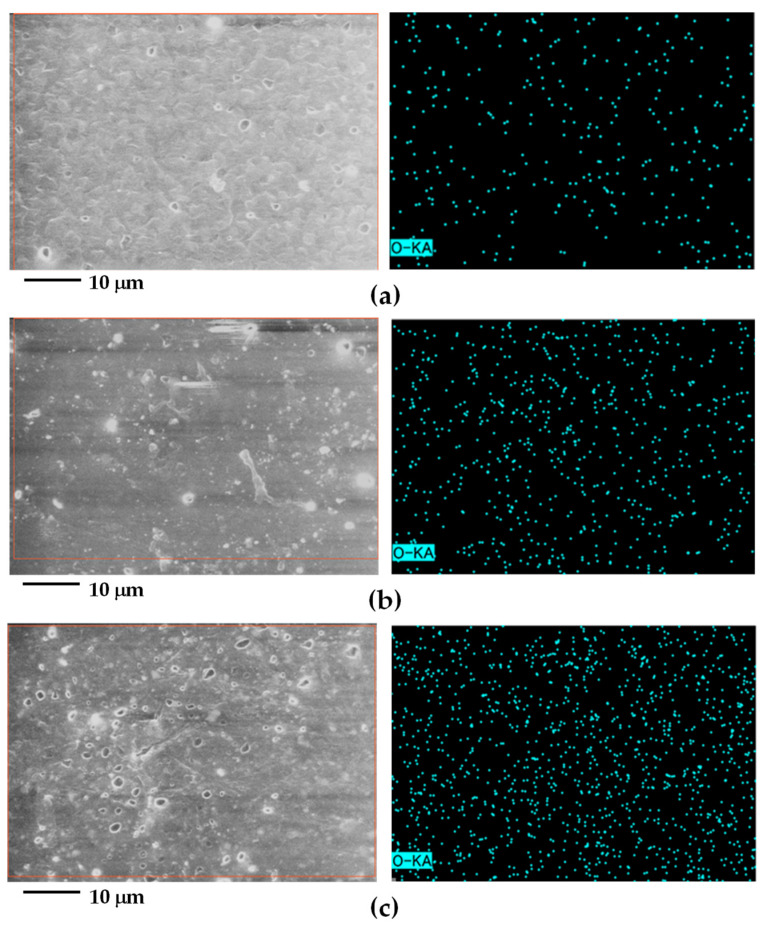
SEM images (**left**) and EDS-mapping oxygen element (**right**) of surface morphology of PPCP9 with ultramarine blue masterbatch: (**a**) PPCP9MB0; (**b**) PPCP9MB3; (**c**) MB (PPCP9).

Figure 3 depicts the morphology of PPCP9 at MB0, MB3, and the masterbatch (MB) of PPCP9, aiming to elucidate the distribution and dispersion of the pigment within the colored thermoplastic matrix in this study. The PPCP morphology comprises ethylene-propylene rubber (EPR)-dispersed particles within the polypropylene (PP) matrix [17], as depicted in Figure 3a (left). It can be noted that oxygen detected in PPCP9MB0 (Figure 3a, right) due to either the conductive layer, oxidative degradation, or erroneous because of low X-ray fluorescence, self-absorption, and detector efficiency [15,18,30]. Hence, the observation of pigment particle dispersion (with a particle size of approximately less than 2 μm) is challenging. Figure 3b,c showcase SEM images and EDS mapping of PPCP9MB3 and the masterbatch of PPCP9. Oxygen mapping exhibited a well-distributed and dispersed pattern, evident in Figure 3b,c for PPCP9MB3 and the masterbatch, respectively. The levels of oxygen mapping increased with the rise in pigment content from MB3 to high concentrations in the masterbatch. These findings suggest that the pigment is effectively distributed and dispersed throughout the polymer matrix [28]. Notably, the EDS mapping did not reveal any significant signs of pigment agglomeration or aggregation.

### 3.2. Rheological Behavior of Thermoplastics Incorporated with Masterbatch

**Figure 4:**
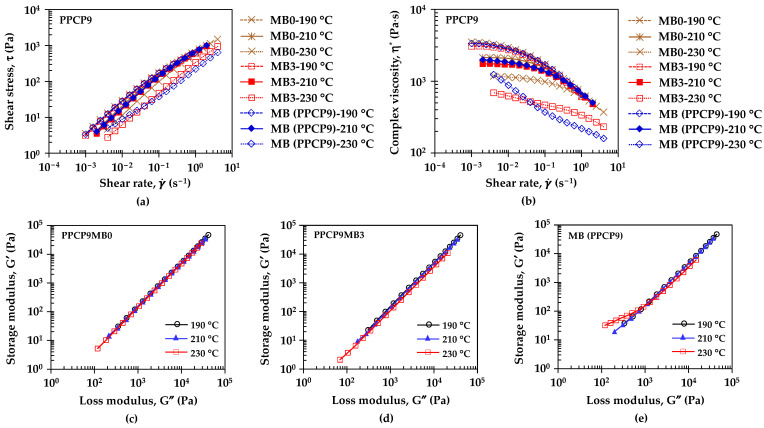
Effect of temperatures on flow curves of PPCP with MB0, MB3, and MB (PPCP9): (**a**) Shear stress; (**b**) Complex viscosity; and Cole-Cole Plots of modulus: (**c**) PPCPMB0; (**d**) PPCPMB3; (**e**) MB (PPCP9).

**Figure 5:**
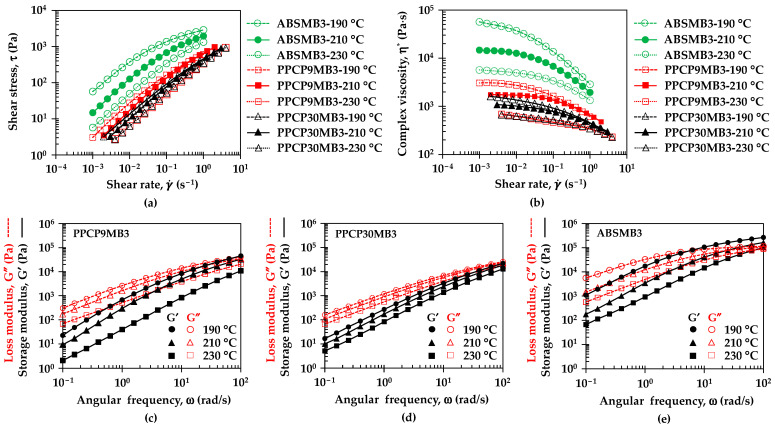
Effect of temperatures on flow curves of PPCP9, PPCP30, and ABS with MB3: (**a**) Shear stress; (**b**) Complex viscosity; and the plot of storage modulus and loss modulus vs. angular frequency: (**c**) PPCP9MB3; (**d**) PPCP30MB3; (**e**) ABSMB3.

Figure 4 and Figure 5 illustrate the rheological properties of neat polymers and colored thermoplastics to understand flow behavior, viscoelasticity, and microstructure such as the state of dispersion in the filled composite dispersion [12,16,31,32,33]. Figure 4a,b show the effect of temperature on the flow curves, i.e., shear stress and complex viscosity as a function of shear rate of colored thermoplastic PPCP9. The measurement temperatures were set at 190, 210, and 230 °C related to the compounding temperatures. These flow curves exhibited non-Newtonian behavior recognized in shear thinning or pseudoplastic [31,33]. The flow curves were fitted to the power-law model by $\tau =K{\dot{\gamma }}^{n}$ where τ is shear stress, K is the consistency index which is equal to the viscosity at 1 s^−1^, $\dot{\gamma }$ is strain rate, and n is the power law index [31,34]. Table A1 summarizes parameters from the power law model of the colored thermoplastics estimated by power regression of the log of shear stress and log of strain rate curves. Shear stress (τ) and complex viscosity (η^*^) of PPCP9MB0, PPCP9MB3, and the masterbatch decreased when increasing temperatures. The flow curves exhibited similar behavior at temperatures of 190 and 210 °C while showing a variation at 230 °C as presented in Figure 4a,b. The power law index (*n*) in Table A1 confirmed the shear thinning of PPCP, and with the incorporation of masterbatch. At low shear rates, polymer melt tended to maintain an irregular order resulting in polymer molecule or pigment particle interactions with a high zero-shear viscosity at 190 and 210 °C in Figure 4b. When shear rate or shear stress is applied high enough to overcome the interaction, polymer melt can stretch and align with the flow, and the pigment particles can rearrange or reorganize [34]. This behavior implied an arrangement of microstructure as well as a breaking down of aggregated structures to primary pigment particles resulting in in the achievement of good pigment dispersion [16,34]. The shear thinning of the PPCP9 materials slightly shifted close to the Newtonian flow indicated by increasing n values at higher temperatures. The consistency index (K) can be referred to as the overall viscosity, which was approximately 210–870 Pa·s for neat PPCP9, the colored materials, and the masterbatch. At the reference temperature of 210 °C, K and n values of the colored PPCP9 did not change with the masterbatch contents or compounding conditions as summarized in Table A1. The Cole–Cole plots of storage modulus (G′) and loss modulus (G″) were applied to distinguish homogeneous and heterogeneous polymer compounds and the dependent of the processing conditions [35]. Figure 4c,d display the Cole–Cole plots of G′ and G″ of PPCP9MB0 and PPCP9MB3, respectively. The curves obtained at different temperatures almost completely coincided, indicating that the melt microstructure of neat PPCP9MB0 and PPCPMB3 were stable and independent of temperature as demonstrated in the research of Xu et al. [35]. Hence, the microstructure and pigment dispersion of the colored PPCP materials were comparable when varying masterbatch contents, screw speeds, and die temperatures. Nevertheless, the shear stress of the PPCPMB3 and the masterbatch decreased when the temperature was set at 230 °C. In addition, the viscosity of PPCPMB3 and the masterbatch significantly dropped when increasing shear rates as compared to PPCPMB0. Notably, the viscosity of the PPCP9MB3 at 230 °C kept similar flow behavior as the lower temperatures assuming that the pigment dispersion and the microstructure were identical. On the contrary, the microstructure of the masterbatch depended on temperatures as shown by the deviation in the Cole–Cole plots in Figure 4e. It was considered that the masterbatch with high pigment contents contained a dispersing agent to improve flow ability and pigment dispersion. The melt microstructure of the masterbatch was unstable and underwent particle aggregation or agglomeration and limited pigment dispersion when compounding at high masterbatch content and high temperature for PPCP9 [12].

Figure 5a,b illustrates the flow curves of colored thermoplastics, PPCP9MB3, PPCP30MB3, and ABSMB3 at different temperatures. All colored thermoplastics at MB3 exhibited shear thinning profiles. Overall complex viscosities and the K values in Table A1 presented the order at high to low values from ABS, PPCP9, then PPCP30, which can be related to molecular weight (MW) of the thermoplastics, higher MW shows higher viscosity [31]. It can be noted that the higher MW of ABS and PPCP9 incurred higher shear stress during compounding than PPCP30. The K values decreased, and the matrix flow ability was increased when increasing temperatures. The literature reports that the dispersion of pigment improved with the viscosity reduction [16]. From Table A1, the n value of ABSMB3 increased when increasing temperature, which shear thinning behavior decreased and developed the zero-shear viscosity as shown in Figure 5b. Therefore, the melt microstructure and the pigment dispersion of ABS would develop when increasing the temperature. However, high temperatures may deteriorate microstructure as well as limit pigment dispersion [12]. The relationship between storage modulus and loss modulus as a function of angular frequency for viscoelastic properties of the colored thermoplastics with MB3 is presented in Figure 5c–e. ABS showed the highest viscoelastic properties, both G′ and G″, which indicated the highest molecular entanglement as compared to PPCP9 and PPCP30 [31]. At higher frequencies, ABSMB3 exhibited solid-like behavior indicated by G′ > G″ that would restrict molecular movement and limit pigment dispersion [12,31,34]. Kozlowska et al. reported that modified pigment masterbatch affected the tendency of the filler to aggregate leading to the higher values of G′ and G″ [12]. The viscoelastic properties of PPCP9MB3, PPCP30MB3, and ABSMB3 decreased when increasing temperature, leading to a reduction of pigment aggregation that corresponded results in [16].

### 3.3. Thermal Properties and Crystallization Behavior of Semicrystalline Polypropylene Copolymer

**Figure 6:**
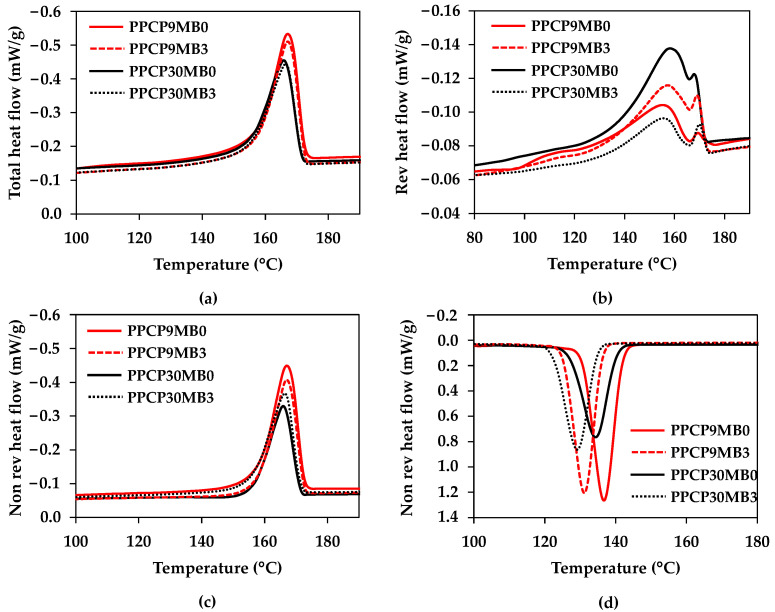
DSC thermograms (endothermic up) of PPCP9 and PPCP30 with MB0 and MB3: (**a**) Total heat flow heating cycle; (**b**) Reversible heat flow heating cycle; (**c**) Nonreversible heat flow heating cycle; (**d**) Nonreversible heat flow cooling cycle.

The effects of masterbatch and compounding conditions on the thermal properties of semicrystalline PPCPs were carried out by modulated differential scanning calorimetry (MDSC). Figure 6 illustrates DSC thermograms from total heat flow, reversible heat flow, and nonreversible heat flow of PPCP9 and PPCP30 at MB0 and MB3. Endothermic peaks of melting temperatures at all heat flows were similarly of melting temperature of polypropylene [17] between PPCP9 and PPCP30 but were different in intensities as shown in Figure 6a–c. It was due to the crystallization ability of the difference MFR as depicted in Figure 6d for the crystallization temperature of each PPCP. Exothermic peaks of PPCP9 display at higher temperatures and higher intensities indicated higher crystallization than PPCP30. Table 3 summarizes the thermal properties of PPCP9 and PPCP30 incorporated with the different contents of masterbatch and various compounding conditions.

From the total heat flow, the melting temperature (T_m Total_), and melting enthalpy (ΔH_m Total_) of PPCPs were unchanged when incorporated with the ultramarine blue masterbatch. The melting enthalpy confirmed that PPCP9 with that lower MFR showed higher crystallinity than PPCP30 with higher MFR. The results were attributed to higher MW of PPCP9 as indicated by the complex viscosity, and the higher ability of molecular orientation of PPCP9 as compared to PPCP30 [36]. On the other hand, heat capacity and thermal behaviors from the changing of the heat capacity signal are observed by the reversible heat flow. The two melting temperatures from the reversible heat flow signals (T_m Rev_) imply the different crystal formations in the PPCPs [37,38]. The melting enthalpy of the reversible heat flows (ΔH_m Rev_) can be attributed to the initial crystallinity of the materials related to the manufacturing process and thermal history [39]. The crystallization behavior was considered on the signal of the nonreversible heat flow to inform the crystallinity by the melting of crystal perfection during kinetic crystallization [40]. The melting enthalpy of the nonreversible heat flow (ΔH_m Nonrev_) of the colored thermoplastics was lower than the neat polymers. The melting temperatures at the nonreversible heat flow signals (**T_m Nonrev_**) were unchanged whereas the crystallization temperature of the nonreversible signals (T_c Nonrev_) decreased when incorporated with the masterbatch. Hence, the ultramarine blue did not act as a nucleating agent for PPCPs, which did not influence the mold shrinkage of the colored PPCPs. On the contrary, the changing of either the screw speed or the die temperature at MB3 influenced the crystallization behavior. The ΔH_m Nonrev_ value of PPCPMB3 slightly increased when changing the screw speed to 100 rpm and decreased when setting the die temperature to 190 °C. It was attributed to a difference in molecular orientation from different shear stress when decreasing screw rotation speed and temperature [41]. Thermal properties of the PPCP masterbatches were also investigated as tabulated in Table 3. Triple endothermic peaks were observed around 104, 142, and 166 °C that related to the melting temperature of polyolefin wax, dispersing agents, and PPCP [2,17,42]. The high content of the pigment in the masterbatch significantly decreased the melting enthalpy, ΔH_m Total_ and ΔH_m Nonrev_, indicating the declination of the crystallinity of the PPCP matrix. The inorganic ultramarine blue pigment exhibited less effectiveness as a nucleating agent for semicrystalline polymer [1,6]. In addition, polypropylene copolymer has been known as a heterophasic polymer [17,18]. Crystallization can occur in both PP and EPR phases depending on EPR contents and different crystallization conditions resulting in variations of crystal morphology and crystallinity of PPCPs [17,43]. Further study on the effect of pigments on the PPCP crystallization affinity is challenging. In this research, the crystallinity of PPCP9, PPCP30, and their colored samples will be discussed based on their influence of color spaces in the next section.

### 3.4. Color Spaces of Thermoplastics Incorporated with Masterbatch

#### 3.4.1. Effects of Compounding Conditions on Color Space

**Figure 7:**
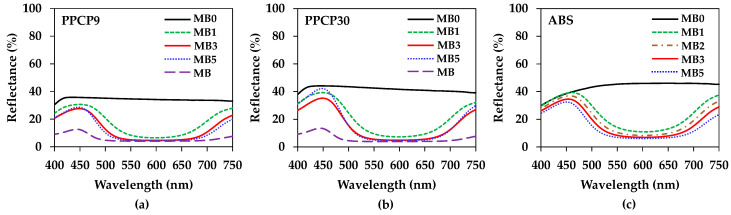
Reflectance spectrums of colored thermoplastics: (**a**) PPCP9; (**b**) PPCP30; (**c**) ABS.

Figure 7 presents the reflectance spectra of thermoplastics colored with an ultramarine blue masterbatch. The inherent color of neat PPCP9, PPCP30, and ABS at MB0 exhibited white reflectance spectra, as depicted in Figure 7a–c, respectively. The translucency of PPCP and the opaque ivory nature of ABS underwent a noticeable shift to blue upon the incorporation of the masterbatch. The white reflectance spectrum of the natural color reflected a blue spectrum at approximately 450 nm, corresponding to the reflectance spectrum of the ultramarine blue pigment, with a slightly reddish hue at 700–750 nm [44]. The intensity of the blue spectrum at 450 nm diminished with increasing masterbatch contents due to lower blue reflection at high opacity [45]. However, at MB1, the reflectance spectrum of the colored thermoplastics exhibited green and yellow hues around 500–600 nm. This shift is attributed to the transformation from natural to blue color, influenced by the thermal history during compounding. Subsequently, the blueness of the colored thermoplastics increased, reducing yellowness with increasing masterbatch content It can be noted that the high pigment content in the masterbatch exhibited the maximum reflectance at 440 nm close to violet and diminished all other color reflectance spectra as shown in Figure 7a,b.

The intensity of the blue reflectance spectrum of the colored thermoplastic was used to calculate the color strength (K/S) based on the Kubelka–Munk equation [14,46,47].

(2) $$K/S=\frac{{\left(1-R\right)}^{2}}{2R}$$

where K is the absorption coefficient, S is the scattering coefficient, and R is the reflectance intensity of the sample.

**Figure 8:**
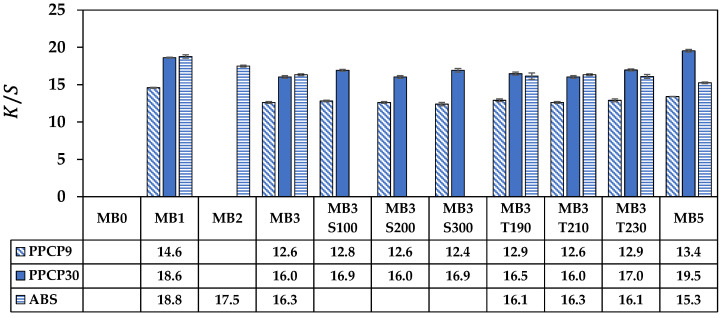
K/S of colored thermoplastics: PPCP9, PPCP30, and ABS with masterbatch.

Figure 8 illustrates the color strength of the compounds. The higher reflectance intensity yielded higher color strength [14,46]. Notably, the intensity of the blue spectrum at 450 nm was linked to the absorption of all spectrums of light except for the blue spectrum, and such reflection is inhibited as the specimen darkened. Hence, the values of color strength slightly decreased with increasing masterbatch content from MB1 to MB3. The color strength in the PPCPs increased whereas it decreased in the ABS samples with the incorporation of MB5. Notably, the color strengths in PPCP30 and ABS are higher than PPCP9, which were higher by about 25% when incorporated with MB3. It was attributed to the opacity of the materials, which was reported in [45]. In addition, the reflectance spectrum was further analyzed and interpreted in numerical color spaces to communicate the color of these thermoplastics.

**Figure 9:**
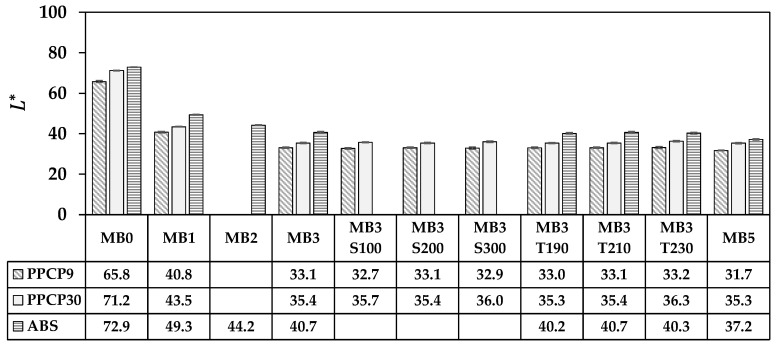
$L^{*}$ of colored thermoplastics: PPCP9, PPCP30, and ABS with masterbatch.

**Figure 10:**
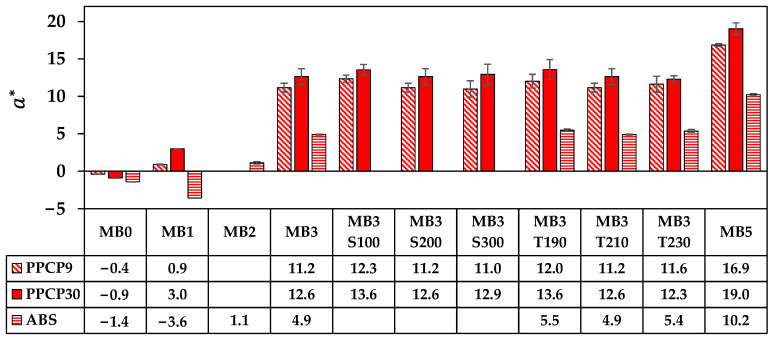
$a^{*}$ of colored thermoplastics: PPCP9, PPCP30, and ABS with masterbatch.

**Figure 11:**
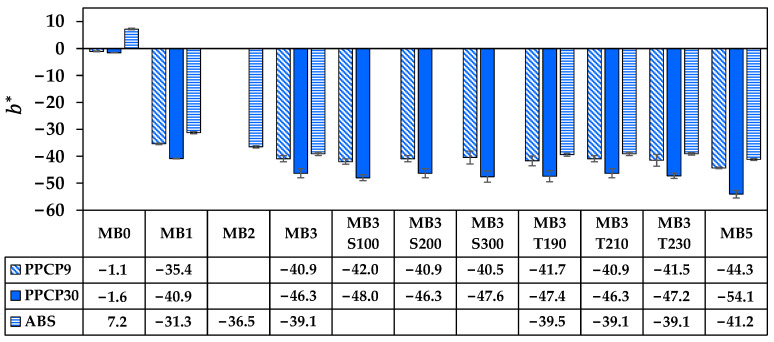
$b^{*}$ of colored thermoplastics: PPCP9, PPCP30, and ABS with masterbatch.

**Figure 12:**
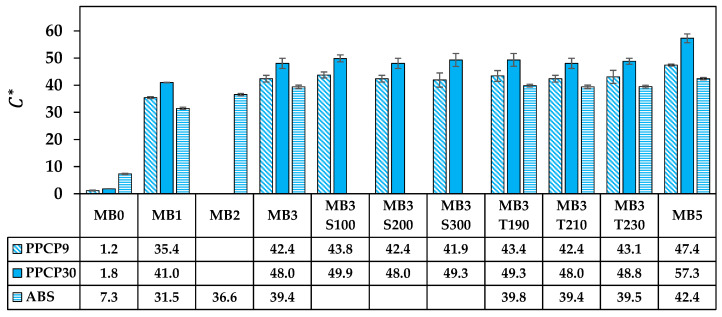
$C^{*}$ of colored thermoplastics: PPCP9, PPCP30, and ABS with masterbatch.

**Figure 13:**
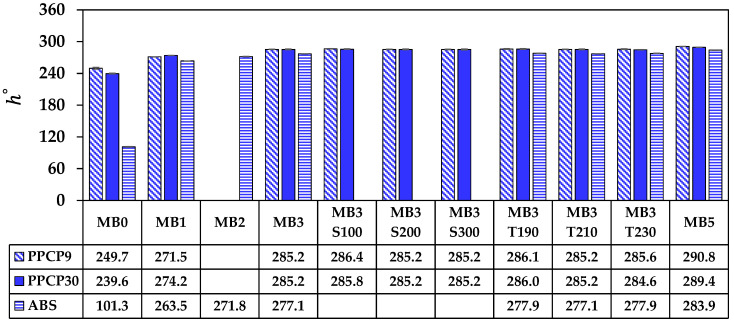
$h^{{}^{\circ}}$ of colored thermoplastics: PPCP9, PPCP30, and ABS with masterbatch.

The rectangular coordinate color space CIELAB has been known as $L^{*}$ for lightness, $a^{*}$ for red and green by positive and negative values, respectively, and $b^{*}$ for yellow and blue by positive and negative values, respectively [14,15,21]. These CIELAB numerical color spaces are calculated from the reflectance spectrum [14,23]. Additionally, the cylindrical coordinate CIELCH is informed lightness, $L^{*}$ value, saturation ($C^{*}$), and hue ($h^{{}^{\circ}}$) of the color [22]. CIELAB and CIELCH are used for quality control and communicating color in polymer products [12,14,15,21,23]. $∆L^{*}$, $∆a^{*}$, and $∆b^{*}$ can be informed of a color deviation between the sample and the standard or the reference, which is calculated as the total color difference known as ${∆E}_{ab}^{*}$ to justify and accept the color of the products [12,21]. The color spaces of thermoplastics incorporated with ultramarine blue masterbatch are displayed with their average values in Figure 9, Figure 10, Figure 11, Figure 12 and Figure 13. All color spaces, $L^{*}$, $b^{*}$, and $C^{*}$ drastically changed with adding MB1 in PPCP9, PPCP30, and ABS. Then, $L^{*}$ decreased while $b^{*}$ and $C^{*}$ increased along with increasing MB as presented in Figure 9, Figure 11 and Figure 12, respectively, whereas $a^{*}$ was a marginal change as in Figure 10. Neat PPCPs have a neutral color of translucent materials while neat ABS shows a yellow of (+$b^{*}$) value from the natural ivory. Hence, the greenness (−$a^{*}$) was indicated by the combination of the blue masterbatch with the ivory ABS in the ABSMB1. The color of the ultramarine blue MB is strongly bluish and reddish with high $C^{*}$ in Figure 12 and confirmed by hue values up to 290 in Figure 13 when increasing MB. The hue values in Figure 13 informed the blue nature color of PPCP9, PPCP30, and the yellow in ABS. Hence, the natural color of the thermoplastic affected their color properties when incorporated with pigments. It can be noted that the number of pigments incorporated with thermoplastics as tabulated in Table A2 were about 0.3, 0.6, and 0.9 wt.% for MB1, MB3, and MB5, respectively. These pigments yielded colorfulness of the ultramarine blue pigment in the colored thermoplastics. The opacity of the pristine PPCPs and ABS depends on their characteristics in translucent and opaque materials. The declination of $L^{*}$ with increasing the contents of the masterbatch revealed an increase in the opacity of the colored thermoplastics as summarized in Table A2. High-MFR PPCP30 samples have higher opacity values than low-MFR PPCP9. It was the difference in the crystallinity of PPCPs, in which the crystallinity of PPCP30 was lower than PPCP9. Hence, the PPCP30 has more light reflection indicated by higher reflectance intensity as compared to PPCP9 resulting in higher color strength and color spaces in the PPCP30 incorporated with masterbatch [14,44]. It is interesting to note that the higher opacity of PPCP30 and ABS exhibited higher color strength 15–45% as compared to PPCP9 at the similar content of the masterbatch.

**Figure 14:**
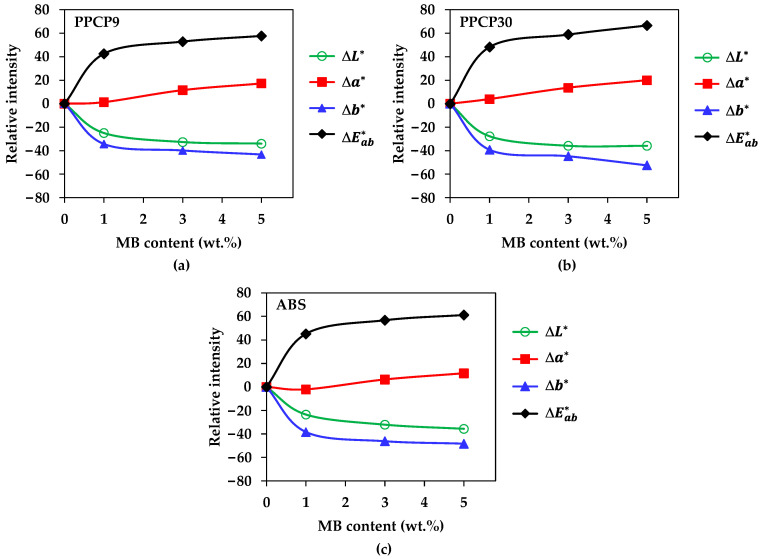
Color differences of colored thermoplastics: (**a**) PPCP9; (**b**) PPCP30; (**c**) ABS.

The color differences at different contents of the masterbatch as compared to the neat polymers are illustrated in Figure 14a–c for PPCP9, PPCP30, and ABS, respectively. ${∆L}^{*}$ and $∆b^{*}$ of all colored thermoplastics showed a negative value, which informed blue deepened in the colored thermoplastics. On the other hand, ${∆a}^{*}$ moved to positive values and became redder from the reddish in the pigment. It can be noted that this blue reddish pigment induced a negative value of $∆a^{*}$ indicating green in ABSMB1 as presented in Figure 14c. ${∆E}_{ab}^{*}$ for the total color difference increased, which confirmed strong color changes in PPCP9, PPCP30, and ABS with ultramarine blue masterbatch. The color differences ${∆L}^{*}$, $∆b^{*}$, and ${∆E}_{ab}^{*}$ were almost unchanged from MB3 to MB5 in the colored materials. It was implied that the blue of colored PPCP9, PPCP30, and ABS thermoplastics has reached saturation at MB3 [7,9]. Nevertheless, the increase in $∆a^{*}$ indicates that the compound turned redder at higher MB contents because of the reddish in the pigment. It can be noted that the lower crystallinity of high-MFR PPCP30 went further in blue by observing an increase of $∆b^{*}$. Therefore, the PPCP30 did not reach saturation at MB5 even though it reached the maximum opacity.

The content of the masterbatch strongly influenced the color spaces of the colored thermoplastics and can be indicated by the color differences. On the other hand, the effects of screw speed and die temperature on color spaces were less noticeable as presented in Figure 9, Figure 10, Figure 11, Figure 12 and Figure 13. The statistical analysis by the analysis of variance (ANOVA) was elucidated to clarify the in-depth investigation of compounding conditions significant differences in the color spaces of the colored thermoplastics.

#### 3.4.2. Statistical Analysis of the Effects of Compounding Conditions on Color Space

The analysis of variance (ANOVA) was conducted to identify terms significantly impacting color space. ANOVA is a statistical method employed to ascertain significant differences among the means of multiple groups [48]. It is frequently utilized to test hypotheses concerning the relationship between an independent variable and a dependent variable. Through the single-factor (one-way) ANOVA design experiment, the study discerns the terms within the experiment that exert a significant influence by masterbatch contents and compounding conditions employed to assess the color spaces, $L^{*}$, $a^{*}$, $b^{*}$, $C^{*}$, and $h^{{}^{\circ}}$, which are displayed as dependent or response variables. The one-way ANOVA is detailed in Appendix A. The experimental design, formulated using statistical techniques, is presented in Table 2, and color space data are outlined in Supplementary Tables S1–S3.

The statistical analysis, employing one-way ANOVA by the Minitab software (Version 17) on color space, is discussed for each thermoplastic material. Overall, the experiment provides a lucid and precise depiction of the material components, as summarized in Table 4. The *p*-value statistics indicate that terms with a *p*-value less than 0.05 are significant to the response variable [48], underscoring those terms with low *p*-values, such as 0.00 in Table 4, possess a statistically significant impact on color space. The adjusted R-square (R-sq (adj)) for all experiments on color space exceeds 95% and up to 100%, signifying acceptability. This indicates that the data for response fit well within the developed models [48]. Consequently, the linear regression model employed in the analysis proves adequate, demonstrating a robust predictive capability within the experiment’s range, as indicated by high values of the predicted R-square (R-sq (pred)) [48]. The Fisher least significant difference (LSD) is used to perform pairwise comparisons of groups means after performing the significant differences from the one-way ANOVA. Fisher pairwise comparisons for the one-way ANOVA using the grouping information table to determine whether the mean difference between any pair of groups is statistically significant. The group column contains letters that group the factor levels. Groups that do not share a letter have a mean difference that is statistically significant [49].

Fisher pairwise comparisons for one-way ANOVA by the LSD method at 95% confidence were utilized to identify the MB, screw speed, and die temperature factors significantly affecting the means or averages of color spaces of the colored thermoplastics by the group columns with results tabulated in Table A3, Table A4, Table A5, Table A6, Table A7, Table A8, Table A9, Table A10, Table A11, Table A12, Table A13, Table A14, Table A15, Table A16 and Table A17. The results can be interpreted through “means that do not share a letter are significantly different” [49].

Table A3, Table A4, Table A5, Table A6 and Table A7 show the grouping information of the color spaces of PPCP9. The results revealed the different letters of grouping indicated that masterbatch has a significant effect on all color spaces. In addition, the ANOVA results for the die temperature and screw speed model indicated that LSD groups were not significant in $L^{*}$, $b^{*}$, and $C^{*}$ when the masterbatch was held constant at MB3. On the contrary, it was found that the screw speed of 100 rpm has statistically significant on $a^{*}$ and $h^{{}^{\circ}}$ as shown in Table A4 and Table A7. The statistical analysis of PPCP9 can conclude that the color spaces are significantly changed by increasing the masterbatch contents. The performance of $L^{*}$, $b^{*}$, and $C^{*}$ remained consistent while keeping MB content constant at 3 wt.%. Hence, we can adjust the die temperature between 190–230 °C and the screw speed between 100–300 rpm without a significant impact on $L^{*}$, $b^{*}$, and $C^{*}$. Nevertheless, the sample would slightly increase in $a^{*}$ and $h^{{}^{\circ}}$ when setting the screw speed at 100 rpm. It was attributed to low interaction between pigment and the polymer matrix and lack of pigment deagglomeration due to differences in rheological and crystallization behaviors as aforementioned.

The Fisher LSD method by one-way ANOVA determining the significance on color spaces of PPCP30 is presented in Table A8, Table A9, Table A10, Table A11 and Table A12. The results demonstrated that all color spaces significantly change according to MB contents. It was noted that the lightness of the PPCP30 was saturated with the MB up to 3 wt.% that $L^{*}$ did not change at MB5. When MB content is maintained at 3 wt.%, based on the confidence interval values, the changing of the screw speed and the die temperature show no significant differences, indicating similar performance of $a^{*}$, $b^{*}$, and $C^{*}$. Therefore, we can vary the die temperature between 190–230 °C and the screw speed between 100–300 rpm without a significant impact $a^{*}$, $b^{*}$, and $C^{*}$ as presented in Table A9, Table A10 and Table A11. On the contrary, the die temperature exhibited statistical significance on $L^{*}$ and $h^{{}^{\circ}}$ when setting at MB3 constantly as depicted in Table A8 and Table A12. It was due to the changing of shear viscosity and molecular entanglement affecting pigment dispersion of high-MFR PPCP30 when setting lower or higher die temperatures [16]. The color of PPCP30MB3 was slightly lower in $h^{{}^{\circ}}$ at 230 °C and alternatively higher in $h^{{}^{\circ}}$ at 190 °C. Therefore, at the die temperature of 210 °C, the screw speed can be adjusted for the PPCPMB3 to meet the general polymer specifications without significant impact on the color spaces.

Table A13, Table A14, Table A15, Table A16 and Table A17 display the Fisher pairwise comparisons results of ABS. As shown in Table 4, the adjusted R-square values were over 99.9%, confirming that less than 0.1% of the total variations in the variables could not be explained by the developed analytic expression. It was observed that color spaces are significantly changed when increased MB content from the grouping results in Table A13, Table A14, Table A15, Table A16 and Table A17. From Table A15 and Table A16, we can conclude that variations in die temperature between 190–230 °C do not significantly impact $b^{*}$ and $C^{*}$. The color spaces at MB3 can be managed by designating the die temperature between 190–230 °C and holding the screw speed constant at 200 rpm for the performance of $b^{*}$ and $C^{*}$. However, the shear viscosity of the ABS is higher than PPCPs. The distribution of the pigment might be low, especially when the die temperature was set at 190 °C. The rheological properties indicated the solid-like behavior at 190 °C that informed high molecular entanglement and restricted polymer mobility resulting in poor pigment distribution [8]. Its lower light reflection translates to slightly lower lightness, redder, and higher hue as compared to the 210 °C as shown in Table A13, Table A14 and Table A17.

Therefore, the statistical analysis by the one-way ANOVA and the Fisher LSD method provides an in-depth understanding of the significance on the color spaces by the MB, screw speed, and die temperature. The die temperature revealed statistical significance on $L^{*}$ and $h^{{}^{\circ}}$ of high-MFR PPCP30 and high melt viscosity of ABS. On the contrary, the screw speed affected $a^{*}$ and $h^{{}^{\circ}}$ of low-MFR PPCP9. This information is valuable for elucidating the effects of compounding conditions on the color spaces of thermoplastics with the ultramarine blue masterbatch. Hence, it is interesting to apply statistical analysis in the quality control assurance and processing control on thermoplastic coloration.

### 3.5. Flexural Properties of Injection Molded Colored Thermoplastics

**Figure 15:**
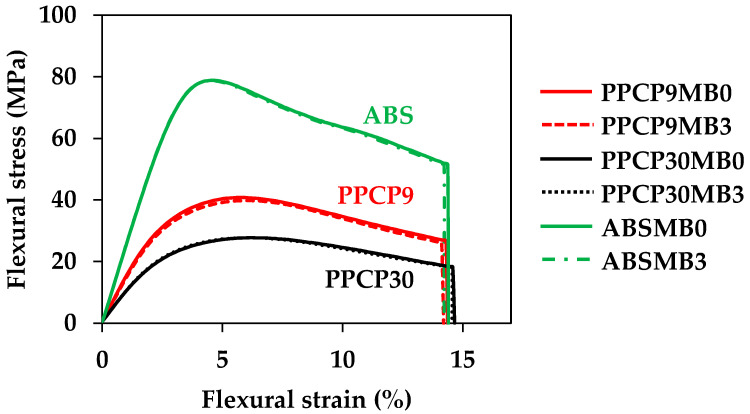
Flexural stress–strain curves of injection molded PPCP9, PPCP30, and ABS with MB0 and MB3.

The introduction of a pigment masterbatch can impact the mechanical properties of filled thermoplastics. To ensure the mechanical performance of injection-molded colored thermoplastics when integrated with the masterbatch, an investigation into their flexural properties was conducted. Figure 15 displays the flexural stress–strain curves of PPCP9, PPCP30, and ABS at MB0 and MB3. These base polymers are recognized for their toughness, with ABS exhibiting the highest flexural stress, followed by low-MFR PPCP9 and high-MFR PPCP30, showing comparable deformation. Table 5 summarizes the flexural properties of thermoplastics integrated with various masterbatch contents. The inclusion of the masterbatch, along with varying compounding conditions such as die temperatures, did not interfere with the flexural properties of the thermoplastics. The incorporating pigment up to 2 wt.% is considered for mechanical and physical properties retaining when compounding with thermoplastics [7]. Therefore, the flexural properties of these thermoplastics, incorporated with the ultramarine blue masterbatch, are suitable for various applications.

## 4. Conclusions

The ultramarine blue pigment masterbatch was developed for compounding with PPPCPs and ASB. SEM-EDS mapping of oxygen revealed fine dispersion of ultramarine blue pigment in polymer matrix. Rheological properties were clarified the optimum conditions on pigment dispersion. The masterbatch contents and screw speeds did not influence rheological properties confirming the similarity in pigment dispersion. At the stage of pigment dispersion, both low-MFR PPCP and high-MFR PPCP can operate at the die temperature of 190–230 °C whereas ABS should avoid processing at the temperature of 190 °C. It can be noted that color strength depends on PPCP crystallinity and opacity of the products as well as color spaces. The masterbatch contents drastically decreased lightness while increasing blue and red upon the ultramarine blue hue. The masterbatch content of about 3 wt.% reached the saturation of blueness and lightness. The Fisher pairwise comparisons for one-way ANOVA have clarified that the screw speed of 100 rpm has statistical significance on increasing redness and hue of low-MFR PPCP. On the other hand, the die temperature has a statistical impact on changing the lightness and hue of high-MFR PPCP and ABS. The ultramarine blue pigment masterbatch did not interfere with the flexural properties of these colored thermoplastics. This comprehensive approach not only advances our understanding of the interplay between masterbatch formulation, compounding conditions, and color properties in thermoplastics but also provides valuable insights for optimizing processes and ensuring the durability and functionality of colored thermoplastic materials.

## Figures and Tables

**Table 1:** Physical and thermal properties of thermoplastics and masterbatch.

Material	Symbol	MFR ^1^ (g/10 min)	T_g_^2^ (°C)	T_m_^2^ (°C)	T_d_^3^ (°C)	Residual ^3^ (%)
PPCP (AW564)	PPCP9	9	N/A ^4^	167.8	425.0	0.06
PPCP (PP8285E1)	PPCP30	30	N/A	167.4	423.0	0.09
ABS (100-322)	ABS	15	104.6	-	408.0	1.29
MB of PPCP9	MB (PPCP9)	N/A	N/A	144.0, 167.0	408.3	15.72
MB of PPCP30	MB (PPCP30)	N/A	N/A	142.1, 165.5	398.1	16.80
MB of ABS	MB (ABS)	N/A	99.0, 105	142.3	395.6	18.71

^1^ Material data sheet from the manufacturers (PPCP at 230 °C, 2.16 kg, ABS at 220 °C, 10 kg). ^2^ Characterize by differential scanning calorimeter from total heat flow. ^3^ Characterize by thermogravimetric analyzer, T_d_ at 5 wt.% mass loss, residual at 550 °C. ^4^ Not applicable (N/A).

**Table 2:** Formulations, conditions, and designation of polymer compounded with masterbatch in a twin-screw extruder.

Parameter	Polymer (wt.%)	MB (wt.%)	Die Temperature (°C)	Screw Speed (rpm)	Designation
Masterbatch (MB)	100	0	210	200	MB0
	99	1	210	200	MB1
	97	3	210	200	MB3
	95	5	210	200	MB5
Screw speed (S)	97	3	210	100	MB3S100
	97	3	210	200	MB3S200
	97	3	210	300	MB3S300
Temperature (T)	97	3	190	200	MB3T190
	97	3	210	200	MB3T210
	97	3	230	200	MB3T230

**Table 3:** Thermal properties of PPCP9 and PPCP30 compounds and their masterbatch.

Material	Sample	Total Heat Flow		Reversible Heat Flow		Nonreversible Heat Flow		
		ΔH_m Total_ (J/g)	T_m Total_ (°C)	ΔH_m Rev_ (J/g)	T_m Rev_ (°C)	ΔH_m Nonrev_ (J/g)	T_m Nonrev_ (°C)	T_c Nonrev_ (°C)
PPCP9	MB0	85.8	167.4	22.4	157.7, 169.1	66.5	167.4	137.0
	MB1	83.9	167.4	23.6	156.7, 170.1	62.2	167.3	133.0
	MB3	82.0	167.1	21.3	157.3, 169.0	62.3	167.0	131.2
	MB5	82.9	167.1	18.7	157.6, 169.1	63.6	167.0	132.7
	MB3S100	85.8	166.9	16.5	155.8, 170.3	66.0	166.9	130.8
	MB3S300	85.3	166.8	14.6	155.0, 169.8	66.7	166.7	130.8
	MB3T190	89.4	167.4	34.3	160.0, 167.8	57.8	167.5	133.6
	MB3T230	85.1	166.5	28.8	158.7, 168.9	56.7	166.5	131.7
	Masterbatch	70.4	104.6, 144.4, 167.0	23.2	145.3, 156.4, 169.7	47.9	104.1, 142.7, 166.9	124.6, 139.7
PPCP30	MB0	72.4	165.9	27.5	158.2, 167.8	46.6	165.9	134.4
	MB1	68.9	166.2	11.5	155.0, 169.2	54.6	166.2	133.2
	MB3	70.7	166.5	11.2	155.3, 169.9	55.4	166.4	129.2
	MB5	68.6	166.2	15.3	156.1, 168.8	51.7	166.1	132.1
	Masterbatch	60.3	103.7, 142.6, 166.2	11.4	144.6, 153.1, 169.6	45.4	103.9, 142.2, 166.1	123.0, 137.5

**Table 4:** Analysis of variance of color spaces of colored thermoplastics.

Material	Degree of Freedom	Color Space	*p*-Value	R-sq (adj)	R-sq (pred)
		$L^{*}$	0.00	99.84	99.75
		$a^{*}$	0.00	98.32	98.08
PPCP9	9	$b^{*}$	0.00	98.76	98.08
		$C^{*}$	0.00	98.65	97.91
		$h^{{}^{\circ}}$	0.00	99.69	99.52
		$L^{*}$	0.00	99.93	99.89
		$a^{*}$	0.00	97.35	95.88
PPCP30	9	$b^{*}$	0.00	98.95	98.37
		$C^{*}$	0.00	98.76	98.08
		$h^{{}^{\circ}}$	0.00	99.81	99.70
		$L^{*}$	0.00	99.92	99.87
		$a^{*}$	0.00	99.92	99.87
ABS	7	$b^{*}$	0.00	99.91	99.85
		$C^{*}$	0.00	99.81	99.70
		$h^{{}^{\circ}}$	0.00	100.00	100.00

**Table 5:** Flexural properties of injection molded colored thermoplastics.

Material	Sample	Flexural Modulus (GPa)	Flexural Strength (MPa)	Flexural Strain (%)
PPCP9	MB0	1.46 ± 0.043	40.8 ± 0.010	14.4 ± 0.017
	MB1	1.42 ± 0.001	40.0 ± 0.097	14.3 ± 0.008
	MB3	1.42 ± 0.005	39.8 ± 0.063	14.2 ± 0.026
	MB5	1.42 ± 0.013	39.6 ± 0.233	14.2 ± 0.008
	MB3T190	1.47 ± 0.022	40.6 ± 0.366	14.3 ± 0.149
	MB3T230	1.39 ± 0.001	39.5 ± 0.017	14.3 ± 0.051
PPCP30	MB0	1.01 ± 0.013	27.7 ± 0.185	14.7 ± 0.066
	MB1	0.99 ± 0.005	27.8 ± 0.138	14.6 ± 0.037
	MB3	1.02 ± 0.006	27.9 ± 0.138	14.6 ± 0.161
	MB5	0.98 ± 0.007	27.3 ± 0.069	14.6 ± 0.098
ABS	MB0	2.55 ± 0.015	79.8 ± 0.427	14.4 ± 0.051
	MB1	2.56 ± 0.014	78.5 ± 0.478	14.1 ± 0.057
	MB3	2.57 ± 0.009	78.6 ± 0.305	14.2 ± 0.059
	MB5	2.57 ± 0.042	79.0 ± 1.192	14.3 ± 0.149

## Data Availability

The data presented in this study are available on request from the corresponding author.

## Supplementary Materials

The following supporting information can be downloaded at: https://www.mdpi.com/article/10.3390/polym15244718/s1, Figure S1: EDS mapping elements of pigment in masterbatch PPCP9 after TGA combustion; Figure S2: EDS mapping elements of injection molded PPCP9MB0; Figure S3: EDS mapping elements of injection molded PPCP9MB3; Figure S4: EDS mapping elements of injection molded masterbatch PPCP9; Table S1: Color spaces of PPCP9 with masterbatch for one-way ANOVA; Table S2: Color spaces of PPCP30 with masterbatch for one-way ANOVA; Table S3: Color spaces of ABS with masterbatch for one-way ANOVA.

## Appendix A

**Table A1:** Power law model of thermoplastics incorporated with masterbatch.

Sample	PPCP9						PPCP30						ABS					
	190 °C		210 °C		230 °C		190 °C		210 °C		230 °C		190 °C		210 °C		230 °C	
	K ^1^	n ^2^	K	n	K	n	K	n	K	n	K	n	K	n	K	n	K	n
MB0	855	0.76	789	0.80	600	0.84							3836	0.57	2684	0.72	1553	0.81
MB1			760	0.82					474	0.81					2838	0.73		
MB3	837	0.77	744	0.82	325	0.85	494	0.78	457	0.82	339	0.85	4260	0.57	2929	0.72	1864	0.81
MB5			754	0.83					472	0.82					2856	0.72		
MB3S100			711	0.82					442	0.82								
MB3S300			704	0.84					434	0.83								
MB3T190			762	0.82					446	0.82								
MB3T230			712	0.82					461	0.82								
MB	872	0.77	761	0.81	214	0.71												

^1^ K (Pa·s) is consistency index. ^2^ n is power law index.

**Table A2:** Residual contents and opacity of thermoplastics incorporated with masterbatch.

Sample	Residual ^1^ (%)			Opacity ^2^		
	PPCP9	PPCP30	ABS	PPCP9	PPCP30	ABS
MB0	0.05	0		61	69	85
MB1	0.28	0.28		85	91	98
MB2						99
MB3	0.59	0.57		94	97	99
MB5	0.93	0.90		97	99	100
MB3S100	0.54			94	97	
MB3S300	0.60			95	97	
MB3T190	0.56			94	98	100
MB3T230	0.61			94	98	99

^1^ Residual at 550 °C characterize by thermogravimetric analyzer. ^2^ Opacity measured by spectrophotometer at reflection mode.

### Statistical Analysis Experimental Design

The analysis of variance (ANOVA) constitutes an experimental design method wherein each treatment is regarded as a random variable within the study. This methodology involves executing the experiment in a random order, considering all potential permutations, to ensure that treatment conditions are applied in as uniform an environment as possible. The purpose is to scrutinize the primary effects and interactions of the factors on the response variable of interest.

In this study, one-way ANOVA is employed as an experimental design to establish the relationship between process responses and input parameters. The independent variables are utilized to represent the response function. This approach enables a thorough examination of the primary effects and interactions of the factors on the response variable of interest, as expressed below [48]:

(A1) $$y_{ij}=\mu +\tau_{i\;}+\epsilon_{ij\;}\{i=1,\;2,\ldots ,a\;j=1,2,\ldots ,n\}$$

where

$y_{ij}$ is a linear function of the model parameters.

μ is an overall mean.

$\tau_{i}$ is a parameter unique to the treatment called the treatment effect.

$\varepsilon_{ij}$ is a random error component that encompasses all other sources of environmental variability, including differences between experimental units and factors that arise from uncontrolled variables.

The Fisher least significant difference (LSD) method, attributed to Fisher, maintains control over the error rate for each specific pairwise comparison but does not extend that control to the experiment-wise or family error rate. This approach relies on the utilization of the t-statistic, as articulated in [48] as follows:

(A2) $$LSD=t_{\frac{\alpha }{2},N-a}\sqrt{\frac{2{Ms}_{E}}{n}}$$

where we could construct a set of 100(1 − α) percent confidence intervals for all pairs of means.

N−a is a degree of freedom error term in the model.

${Ms}_{E}$ is mean square error of the mean.

n is sample for experiment.

To apply the Fisher LSD procedure, we simply compare the observed difference between each pair of averages to the corresponding LSD value. If $\left|\bar{y_{i}}-\bar{y_{j}}\right|$ greater than the LSD, you can infer that there is a significant difference between the population means $u_{i}$ and $u_{j}$ [48].

**Table A3:** Fisher pairwise comparisons by one-way ANOVA of $L^{*}$ Parameter of PPCP9 Compounds.

Fisher Pairwise Comparisons			
Grouping Information Using the Fisher LSD Method and 95% Confidence			
Material	N	Mean	Grouping
PPCP9MB0	3	65.750	A
PPCP9MB1	3	40.757	B
PPCP9MB3T230	3	33.153	C
PPCP9MB3T210	3	33.077	C
PPCP9MB3S200	3	33.077	C
PPCP9MB3	3	33.077	C
PPCP9MB3T190	3	33.030	C
PPCP9MB3S300	3	32.920	C
PPCP9MB3S100	3	32.720	C
PPCP9MB5	3	31.7267	D

**Table A4:** Fisher pairwise comparisons by one-way ANOVA of $a^{*}$ Parameter of PPCP9 Compounds.

Fisher Pairwise Comparisons			
Grouping Information Using the Fisher LSD Method and 95% Confidence			
Material	N	Mean	Grouping
PPCP9MB5	3	16.853	A
PPCP9MB3S100	3	12.347	B
PPCP9MB3T190	3	12.027	B C
PPCP9MB3T230	3	11.623	B C
PPCP9MB3T210	3	11.160	C
PPCP9MB3S200	3	11.160	C
PPCP9MB3	3	11.160	C
PPCP9MB3S300	3	10.980	C
PPCP9MB1	3	0.8967	D
PPCP9MB0	3	−0.3967	E

**Table A5:** Fisher pairwise comparisons by one-way ANOVA of $b^{*}$ Parameter of PPCP9 Compounds.

Fisher Pairwise Comparisons			
Grouping Information Using the Fisher LSD Method and 95% Confidence			
Material	N	Mean	Grouping
PPCP9MB0	3	−1.0800	A
PPCP9MB1	3	−35.410	B
PPCP9MB3S300	3	−40.47	C
PPCP9MB3T210	3	−40.923	C
PPCP9MB3S200	3	−40.923	C
PPCP9MB3	3	−40.923	C
PPCP9MB3T230	3	−41.50	C
PPCP9MB3T190	3	−41.73	C
PPCP9MB3S100	3	−42.013	C D
PPCP9MB5	3	−44.347	D

**Table A6:** Fisher pairwise comparisons by one-way ANOVA of $C^{*}$ Parameter of PPCP9 Compounds.

Fisher Pairwise Comparisons			
Grouping Information Using the Fisher LSD Method and 95% Confidence			
Material	N	Mean	Grouping
PPCP9MB5	3	47.443	A
PPCP9MB3S100	3	43.790	B
PPCP9MB3T190	3	43.43	B
PPCP9MB3T230	3	43.10	B
PPCP9MB3T210	3	42.420	B
PPCP9MB3S200	3	42.420	B
PPCP9MB3	3	42.420	B
PPCP9MB3S300	3	41.93	B
PPCP9MB1	3	35.420	C
PPCP9MB0	3	1.1500	D

**Table A7:** Fisher pairwise comparisons by one-way ANOVA of $h^{{}^{\circ}}$ Parameter of PPCP9 Compounds.

Fisher Pairwise Comparisons			
Grouping Information Using the Fisher LSD Method and 95% Confidence			
Material	N	Mean	Grouping
PPCP9MB5	3	290.807	A
PPCP9MB3S100	3	286.373	B
PPCP9MB3T190	3	286.057	B C
PPCP9MB3T230	3	285.623	B C
PPCP9MB3T210	3	285.247	C
PPCP9MB3S200	3	285.247	C
PPCP9MB3	3	285.247	C
PPCP9MB3S300	3	285.157	C
PPCP9MB1	3	271.453	D
PPCP9MB0	3	249.683	E

**Table A8:** Fisher pairwise comparisons by one-way ANOVA of $L^{*}$ Parameter of PPCP30 Compounds.

Fisher Pairwise Comparisons			
Grouping Information Using the Fisher LSD Method and 95% Confidence			
Material	N	Mean	Grouping
PPCP30MB0	3	71.2033	A
PPCP30MB1	3	43.4533	B
PPCP30MB3T230	3	36.293	C
PPCP30MB3S300	3	36.013	C D
PPCP30MB3S100	3	35.720	D E
PPCP30MB3T210	3	35.397	E
PPCP30MB3S200	3	35.397	E
PPCP30MB3	3	35.397	E
PPCP30MB5	3	35.347	E
PPCP30MB3T190	3	35.347	E

**Table A9:** Fisher pairwise comparisons by one-way ANOVA of $a^{*}$ Parameter of PPCP30 Compounds.

Fisher Pairwise Comparisons			
Grouping Information Using the Fisher LSD Method and 95% Confidence			
Material	N	Mean	Grouping
PPCP30MB5	3	19.023	A
PPCP30MB3T190	3	13.580	B
PPCP30MB3S100	3	13.550	B
PPCP30MB3S300	3	12.933	B
PPCP30MB3T210	3	12.630	B
PPCP30MB3S200	3	12.630	B
PPCP30MB3	3	12.630	B
PPCP30MB3T230	3	12.297	B
PPCP30MB1	3	2.9867	C
PPCP30MB0	3	−0.92667	D

**Table A10:** Fisher pairwise comparisons by one-way ANOVA of $b^{*}$ Parameter of PPCP30 Compounds.

Fisher Pairwise Comparisons			
Grouping Information Using the Fisher LSD Method and 95% Confidence			
Material	N	Mean	Grouping
PPCP30MB0	3	−1.5800	A
PPCP30MB1	3	−40.8867	B
PPCP30MB3T210	3	−46.343	C
PPCP30MB3S200	3	−46.343	C
PPCP30MB3	3	−46.343	C
PPCP30MB3T230	3	−47.247	C
PPCP30MB3T190	3	−47.40	C
PPCP30MB3S300	3	−47.59	C
PPCP30MB3S100	3	−47.987	C
PPCP30MB5	3	−54.060	D

**Table A11:** Fisher pairwise comparisons by one-way ANOVA of $C^{*}$ Parameter of PPCP30 Compounds.

Fisher Pairwise Comparisons			
Grouping Information Using the Fisher LSD Method and 95% Confidence			
Material	N	Mean	Grouping
PPCP30MB5	3	57.313	A
PPCP30MB3S100	3	49.863	B
PPCP30MB3S300	3	49.32	B
PPCP30MB3T190	3	49.32	B
PPCP30MB3T230	3	48.820	B
PPCP30MB3T210	3	48.04	B
PPCP30MB3S200	3	48.04	B
PPCP30MB3	3	48.04	B
PPCP30MB1	3	40.9933	C
PPCP30MB0	3	1.8333	D

**Table A12:** Fisher pairwise comparisons by one-way ANOVA of $h^{{}^{\circ}}$ Parameter of PPCP30 Compounds.

Fisher Pairwise Comparisons			
Grouping Information Using the Fisher LSD Method and 95% Confidence			
Material	N	Mean	Grouping
PPCP30MB5	3	289.380	A
PPCP30MB3T190	3	285.957	B
PPCP30MB3S100	3	285.763	B
PPCP30MB3T210	3	285.230	B C
PPCP30MB3S200	3	285.230	B C
PPCP30MB3	3	285.230	B C
PPCP30MB3S300	3	285.177	B C
PPCP30MB3T230	3	284.590	C
PPCP30MB1	3	274.180	D
PPCP30MB0	3	239.560	E

**Table A13:** Fisher pairwise comparisons by one-way ANOVA of $L^{*}$ Parameter of ABS Compounds.

Fisher Pairwise Comparisons			
Grouping Information Using the Fisher LSD Method and 95% Confidence			
Material	N	Mean	Grouping
ABSMB0	3	72.8867	A
ABSMB1	3	49.340	B
ABSMB2	3	44.2233	C
ABSMB3T210	3	40.703	D
ABSMB3	3	40.703	D
ABSMB3T230	3	40.263	D E
ABSMB3T190	3	40.150	E
ABSMB5	3	37.187	F

**Table A14:** Fisher pairwise comparisons by one-way ANOVA of $a^{*}$ Parameter of ABS Compounds.

Fisher Pairwise Comparisons			
Grouping Information Using the Fisher LSD Method and 95% Confidence			
Material	N	Mean	Grouping
ABSMB5	3	10.2067	A
ABSMB3T190	3	5.4800	B
ABSMB3T230	3	5.390	B
ABSMB3T210	3	4.8767	C
ABSMB3	3	4.8767	C
ABSMB2	3	1.1200	D
ABSMB0	3	−1.43667	E
ABSMB1	3	−3.5867	F

**Table A15:** Fisher pairwise comparisons by one-way ANOVA of $b^{*}$ Parameter of ABS Compounds.

Fisher Pairwise Comparisons			
Grouping Information Using the Fisher LSD Method and 95% Confidence			
Material	N	Mean	Grouping
ABSMB0	3	7.193	A
ABSMB1	3	−31.293	B
ABSMB2	3	−36.547	C
ABSMB3T210	3	−39.097	D
ABSMB3	3	−39.097	D
ABSMB3T230	3	−39.107	D
ABSMB3T190	3	−39.463	D
ABSMB5	3	−41.170	E

**Table A16:** Fisher pairwise comparisons by one-way ANOVA of $C^{*}$ Parameter of ABS Compounds.

Fisher Pairwise Comparisons			
Grouping Information Using the Fisher LSD Method and 95% Confidence			
Material	N	Mean	Grouping
ABSMB5	3	42.417	A
ABSMB3T190	3	39.840	B
ABSMB3T230	3	39.480	B
ABSMB3T210	3	39.403	B
ABSMB3	3	39.403	B
ABSMB2	3	36.563	C
ABSMB1	3	31.500	D
ABSMB0	3	7.333	E

**Table A17:** Fisher pairwise comparisons by one-way ANOVA of $h^{{}^{\circ}}$ Parameter of ABS Compounds.

Fisher Pairwise Comparisons			
Grouping Information Using the Fisher LSD Method and 95% Confidence			
Material	N	Mean	Grouping
ABSMB5	3	283.927	A
ABSMB3T190	3	277.907	B
ABSMB3T230	3	277.853	B
ABSMB3T210	3	277.110	C
ABSMB3	3	277.110	C
ABSMB2	3	271.760	D
ABSMB1	3	263.467	E
ABSMB0	3	101.303	F
