# Supplementary material for: Influence of Compounding Parameters on Color Space and Properties of Thermoplastics with Ultramarine Blue Pigment
Source: Polymers (Basel). 2023 Dec 15;15(24):4718. doi: 10.3390/polym15244718 (PMC10747715; doi:10.3390/polym15244718)
Supplement: Supplementary file 1 [file polymers-15-04718-s001.zip › polymers-2768227-supplementary.pdf]

Influence of Compounding Parameters on Color Space and Properties of Thermoplastics with Ultramarine Blue Pigment

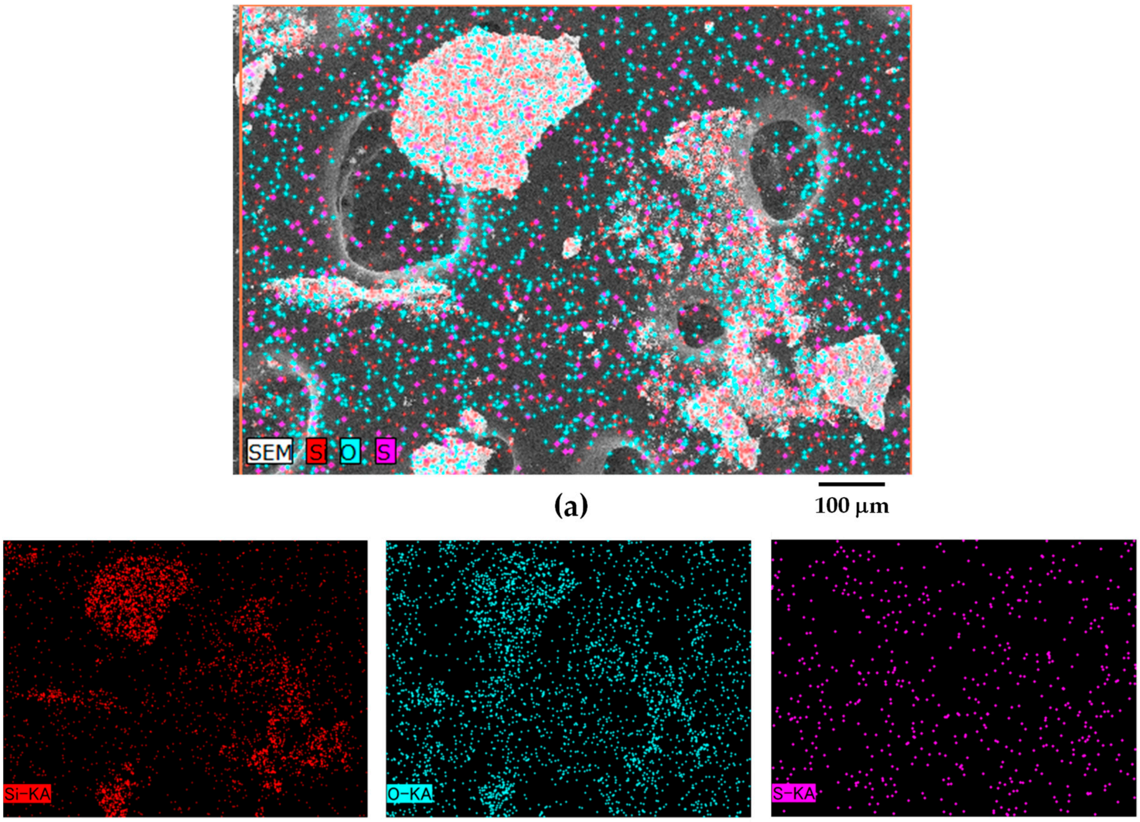

(a)

(b)

Figure S1 EDS mapping elements of pigment in masterbatch PPCP9 after TGA combustion.

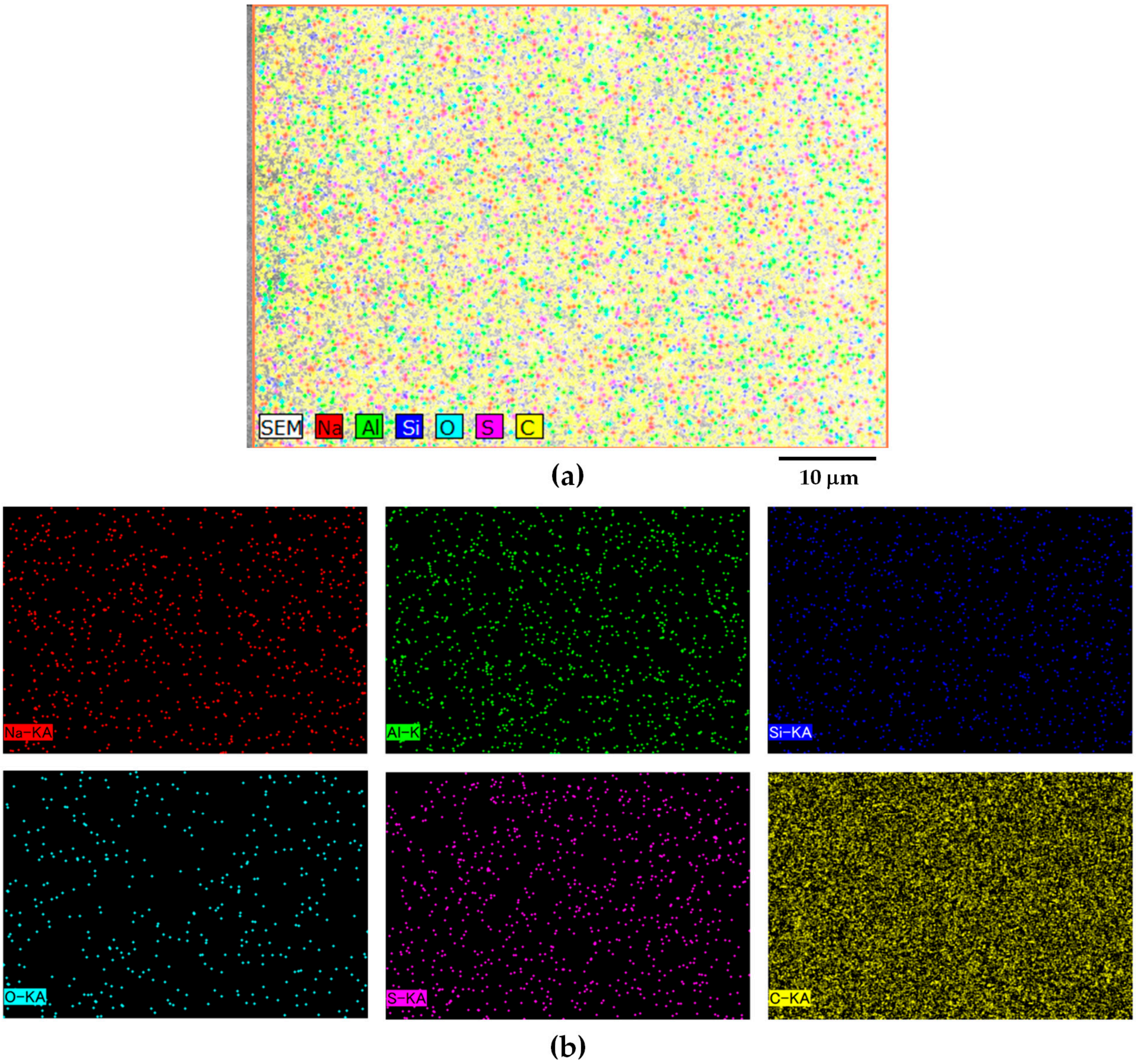

Figure S2 EDS mapping elements of injection molded PPCP9MB0.

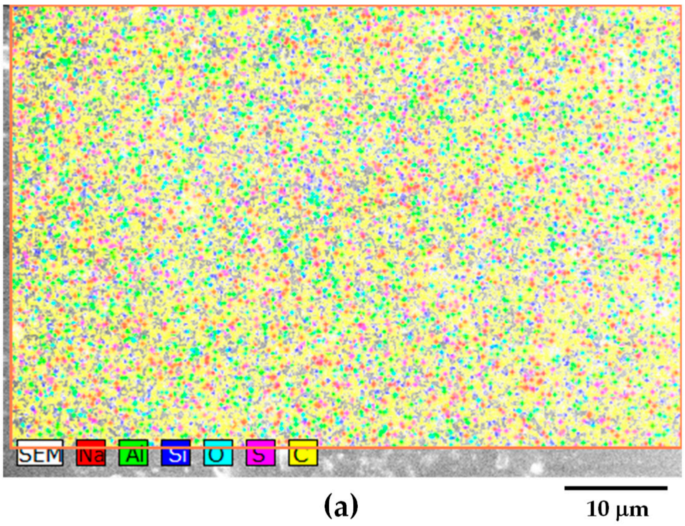

(a)

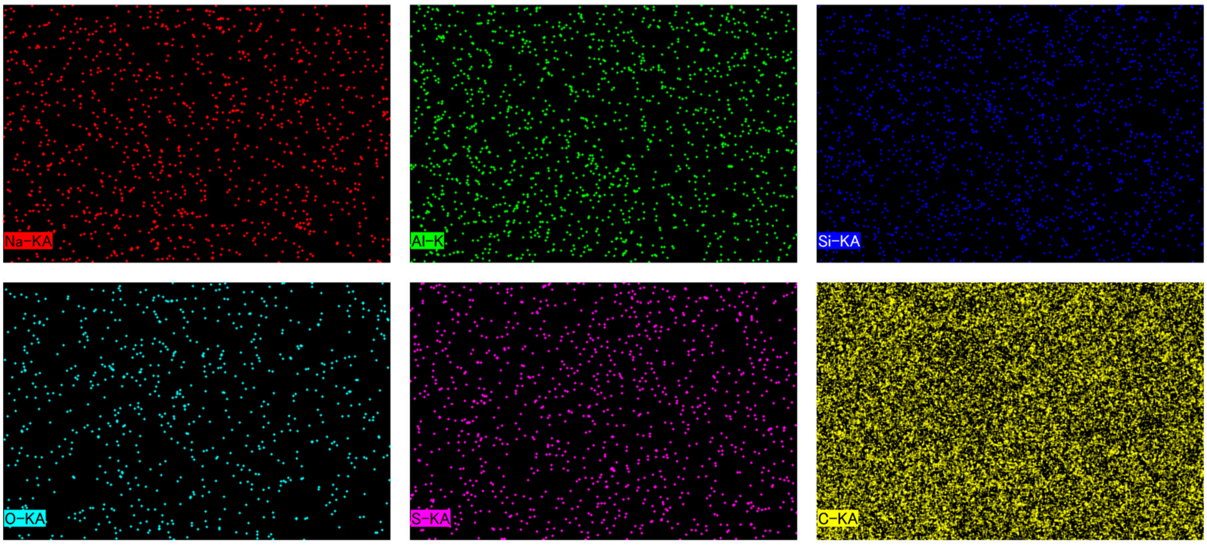

(b)

Figure S3 EDS mapping elements of injection molded PPCP9MB3.

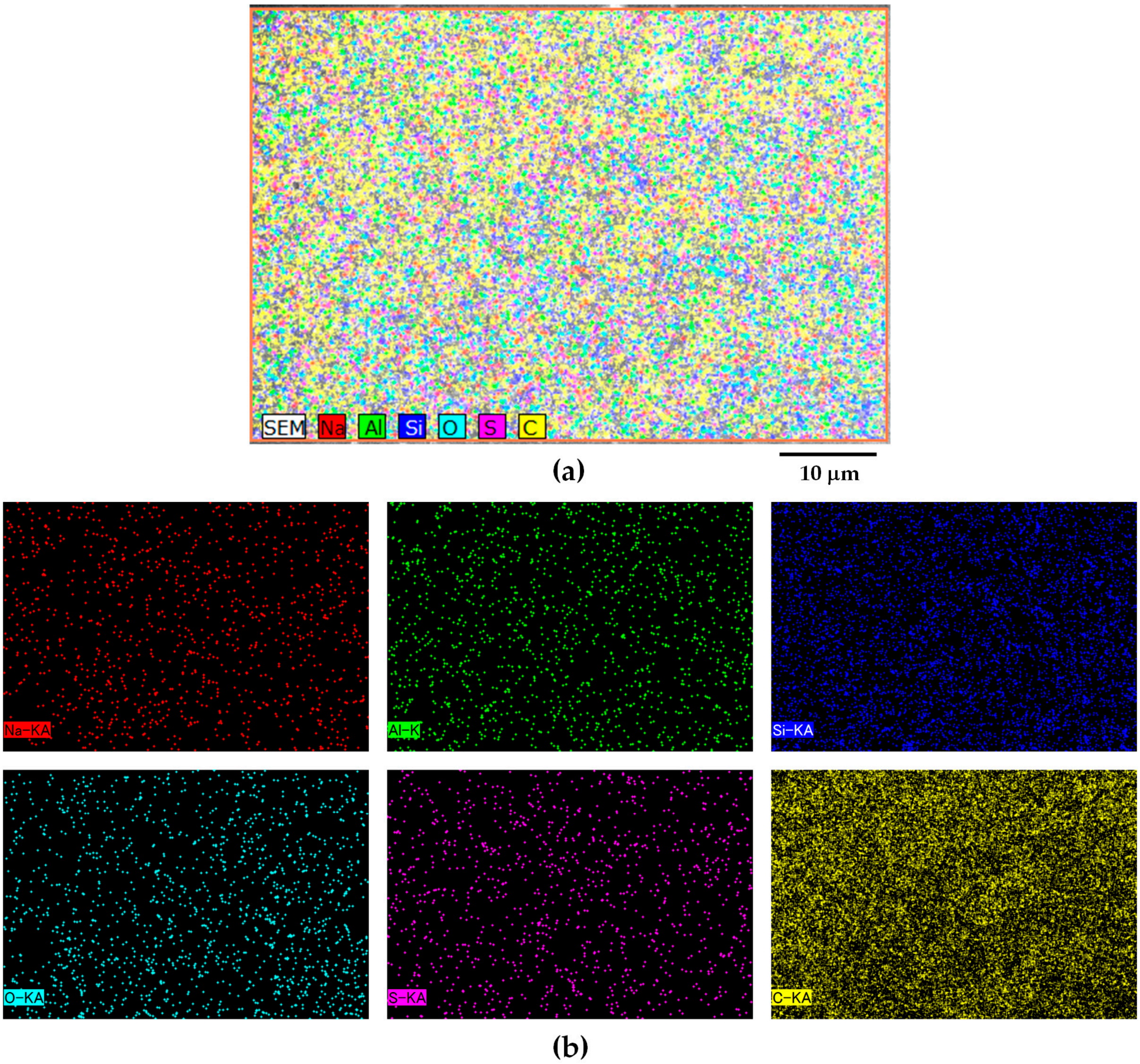

Figure S4 EDS mapping elements of injection molded masterbatch PPCP9.

Table S1 Color spaces of PPCP9 with masterbatch for one-way ANOVA.

PPCP9	L^*	L^*	L^*	a^*	a^*	a^*	b^*	b^*	b^*	C^*	C^*	C^*	h°	h°	h°
MB0	65.5	65.4	66.4	-0.4	-0.4	-0.4	-0.9	-1.2	-1.2	1.0	1.2	1.3	248	251	250
MB1	40.5	40.6	41.2	1.0	0.8	0.9	-35.6	-35.0	-35.6	35.6	35.1	35.6	272	271	271
MB3	32.7	33.2	33.4	10.5	11.4	11.6	-39.7	-41.4	-41.8	41.0	42.9	43.4	285	285	285
MB5	31.9	31.6	31.7	16.7	16.8	17.1	-44.1	-44.2	-44.7	47.2	47.3	47.9	291	291	291
MB3S100	32.3	32.9	32.9	11.8	12.5	12.8	-40.9	-42.2	-42.9	42.6	44.0	44.8	286	286	287
MB3S200	32.7	33.2	33.4	10.5	11.4	11.6	-39.7	-41.4	-41.8	41.0	42.9	43.4	285	285	285
MB3S300	32.3	33.2	33.3	9.7	11.6	11.7	-37.7	-41.7	-42.0	38.9	43.3	43.6	284	286	285
MB3T190	32.6	33.3	33.3	11.0	12.5	12.6	-39.7	-42.6	-43.0	41.2	44.4	44.8	285	286	286
MB3T210	32.7	33.2	33.4	10.5	11.4	11.6	-39.7	-41.4	-41.8	41.0	42.9	43.4	285	285	285
MB3T230	32.6	33.4	33.5	10.4	12.2	12.3	-39.0	-42.6	-43.0	40.3	44.3	44.7	285	286	286

Table S2 Color spaces of PPCP30 with masterbatch for one-way ANOVA.

PPCP30	L^*	L^*	L^*	a^*	a^*	a^*	b^*	b^*	b^*	C^*	C^*	C^*	h°	h°	h°
MB0	71.0	71.2	71.3	-0.9	-0.9	-0.9	-1.6	-1.6	-1.6	1.9	1.8	1.8	240	239	239
MB1	43.5	43.5	43.3	3.0	3.0	3.0	-40.8	-40.8	-41.0	40.9	40.9	41.1	274	274	274
MB3	35.1	35.7	35.4	11.4	13.1	13.4	-44.4	-47.2	-47.5	45.9	49.0	49.3	284	286	286
MB5	35.0	35.6	35.4	18.1	19.4	19.6	-52.4	-54.7	-55.0	55.5	58.1	58.4	289	290	290
MB3S100	35.6	36.0	35.6	12.8	13.7	14.1	-46.8	-48.3	-49.0	48.5	50.2	51.0	285	286	286
MB3S200	35.1	35.7	35.4	11.4	13.1	13.4	-44.4	-47.2	-47.5	45.9	49.0	49.3	284	286	286
MB3S300	35.6	36.4	36.1	11.4	13.6	13.9	-45.2	-48.6	-49.0	46.6	50.4	51.0	284	286	286
MB3T190	35.1	35.6	35.4	12.1	14.2	14.5	-45.0	-48.5	-48.7	46.6	50.6	50.8	285	286	287
MB3T210	35.1	35.7	35.4	11.4	13.1	13.4	-44.4	-47.2	-47.5	45.9	49.0	49.3	284	286	286
MB3T230	35.9	36.6	36.4	11.8	12.6	12.6	-46.0	-47.8	-47.9	47.5	49.5	49.5	284	285	285

Table S3 Color spaces of ABS with masterbatch for one-way ANOVA.

ABS	L^*	L^*	L^*	a^*	a^*	a^*	b^*	b^*	b^*	C^*	C^*	C^*	h°	h°	h°
MB0	73.1	72.7	72.9	-1.4	-1.4	-1.4	7.1	7.5	7.0	7.2	7.6	7.2	102	101	101
MB1	49.2	49.2	49.6	-3.6	-3.6	-3.6	-31.8	-31.2	-30.9	32.0	31.4	31.2	264	263	263
MB2	44.0	44.3	44.3	1.3	1.0	1.1	-37.0	-36.2	-36.5	37.0	36.2	36.5	272	272	272
MB3	40.3	40.7	41.1	5.0	4.9	4.8	-39.8	-38.9	-38.6	40.1	39.2	38.9	277	277	277
MB5	36.9	37.2	37.5	10.4	10.2	10.1	-41.6	-41.1	-40.8	42.9	42.4	42.0	284	284	284
MB3T190	39.7	40.2	40.6	5.6	5.3	5.5	-40.0	-39.0	-39.5	40.4	39.3	39.8	278	278	278
MB3T210	40.3	40.7	41.1	5.0	4.9	4.8	-39.8	-38.9	-38.6	40.1	39.2	38.9	277	277	277
MB3T230	39.9	40.3	40.6	5.6	5.2	5.4	-39.7	-38.8	-38.9	40.1	39.1	39.3	278	278	278
